# Quantitative expression analysis of HHV-6 cell receptor CD46 on cells of human cord blood, peripheral blood and G-CSF mobilised leukapheresis cells

**DOI:** 10.1186/1743-422X-3-77

**Published:** 2006-09-19

**Authors:** Stefanie Thulke, Aleksandar Radonić, Andreas Nitsche, Wolfgang Siegert

**Affiliations:** 1 Charité-Universitätsmedizin Berlin, CCM – Medizinische Klinik m.S. Onkologie/Hämatologie, Charitéplatz 1, 10117 Berlin, Germany; 2 Charité-Universitätsmedizin Berlin, CCM – Medizinische Klinik m.S. Onkologie/Hämatologie, Charitéplatz 1, 10117 Berlin, Germany; 3 Robert Koch Institut, ZBS 1, Nordufer 20, 13353 Berlin, Germany; 4 Charité-Universitätsmedizin Berlin, CCM – Medizinische Klinik m.S. Onkologie/Hämatologie, Charitéplatz 1, 10117 Berlin, Germany

## Abstract

Human herpesvirus-6 (HHV-6) can infect blood cells and thereby may inhibit hematopoietic stem and progenitor cell expansion and differentiation. In this context, it has been discussed if early progenitor cells can be infected by HHV-6. CD46 was identified as one possible cellular surface receptor for HHV-6. The study presented here had been done to get insight into the susceptibility of various leukocyte subpopulations to HHV-6 (including early hematopoietic progenitors) by determining the amount of CD46 molecules expressed on their surfaces. Human cord blood cells, peripheral blood cells and G-CSF mobilised progenitor cells were analysed by flow cytometry. CD46 molecule number per cell was determined and compared to calibration beads conjugated with known ratio of PE per bead. Highest CD46 expression was detected on B- lymphocytes, whereas T-lymphocytes only showed about half of the amount found on B cells. Hematopoietic progenitors also carried CD46 at intermediate levels. Unexpectedly, CD46 expression on progenitors from G-CSF mobilised leukapheresis products was approximately 20% of that found on comparable cells from untreated cord blood. In conclusion, hematopoietic progenitor cells express CD46 on their surface, thereby fulfilling a basic requirement for the susceptibility of HHV-6 infection.

## Findings

Human herpesvirus-6 (HHV-6) was first isolated in 1986 [1]. To date all HHV-6 isolates can be differentiated into the variants HHV-6A and HHV-6B. In early childhood HHV-6B infection causes *exanthema subitum *and febrile illness. After primary infection, HHV-6 persists life-long in host cells and may be reactivated under conditions of immunosuppression, thereby causing various, in some extend life-threatening diseases, including mononucleosis, lymphoid/hematopoietic diseases, myelosuppression, encephalitis, pulmonitis and hepatitis [2]. HHV-6 induced myelosuppression, as occurring after stem cell transplantation, is recognised by leuko- and thrombocytopenia. Moreover, we showed that early HHV-6B infections may contribute to delayed platelet engraftment after stem cell transplantation [3]. There have been evidences for latently HHV-6 infected hematopoietic progenitors reactivating HHV-6 replication within the graft [4,5]. Our own studies showed that HHV-6A as well as HHV-6B are able to infect cord blood (CB) derived mononuclear cells and thereby inhibit *in vitro *expansion of the total cell number and of BFU-e, CFU-GM, as well as CD34^+ ^or CD33^+ ^cells. Contrariwise we could show only less HHV-6 mediated inhibition of CD34^+ ^cell expansion of MACS separated CB CD34^+ ^cells [6]. So far we have had no success to show an infected CD34^+ ^cell. In order to clarify the role of HHV-6 in early hematopoietic stem cell development, we were interested in determining the level of differentiation when blood cells, especially early hematopoietic progenitor cells, became susceptible for HHV-6 infection. We believed that this question might be answered by quantifying HHV-6 membrane receptor CD46. CD46 was identified as cellular surface receptor for HHV-6 in 1999 [7] by interaction with viral glycoprotein complex gH-gL-gQ [8]. CD46 is the cellular receptor for further pathogens: Measles virus, group B adenoviruses [9] and other pathogenic microorganisms [10,11]. It is also known to act as a membrane cofactor for factor-I proteolytic cleavage of C3b and C4b in complement activation. CD46 also affects various cellular activities in response to pathogen or complement binding, and thus influences the host response to infection [12]. Recently, analysis of the short consensus repeat (SCR) regions that comprise most of the extracellular domain of CD46, was shown to have an essential role of SCR 2 and 3 in HHV-6 receptor activity [13].

We analysed samples of peripheral blood (PB) of healthy adults, CB, G-CSF mobilised peripheral blood progenitor cells collected as leukapheresis product (LP), and the HHV-6 infectable cell lines KG-1 and CRF-HSB-2 with regard to CD46 expression. In addition, we analysed CD34^+ ^hematopoietic precursor cells purified by MACS separation (Miltenyi Biotec GmbH, Bergisch Gladbach, Germany). Heparinised blood was diluted 1:10 in FACS lysing Solution (Becton Dickinson, Heidelberg, Germany) to lyse erythrocytes and cells were washed twice in PBS. The cells were stained with the following monoclonal antibodies (mAB) for 15 min at room temperature: R-PE conjugated anti-CD46 (Cymbus Biotechnology LTD, Chandlers Ford, UK) and PerCP conjugated anti-CD45 (Becton Dickinson). To characterise different blood cell types, cells were stained with the following FITC conjugated mAB: anti-CD3, anti-CD8, anti-CD13, anti-CD15, anti-CD19, anti-CD22, anti-CD28, anti-CD33, anti-CD38, anti-CD45, anti-CD65 (Beckman Coulter GmbH, Unterschleissheim-Lohhof, Germany), anti-CD4, anti-CD14, (Becton Dickinson), anti-CD34 (Miltenyi Biotec GmbH). All FACS analyses were performed using the FACSCalibur (Becton Dickinson). Levels of CD46 expression were determined in reference to calibration beads conjugated with a known ratio of PE per bead (QuantiBRITE PE conjugated beads, Becton Dickinson).

**Figure 1:**
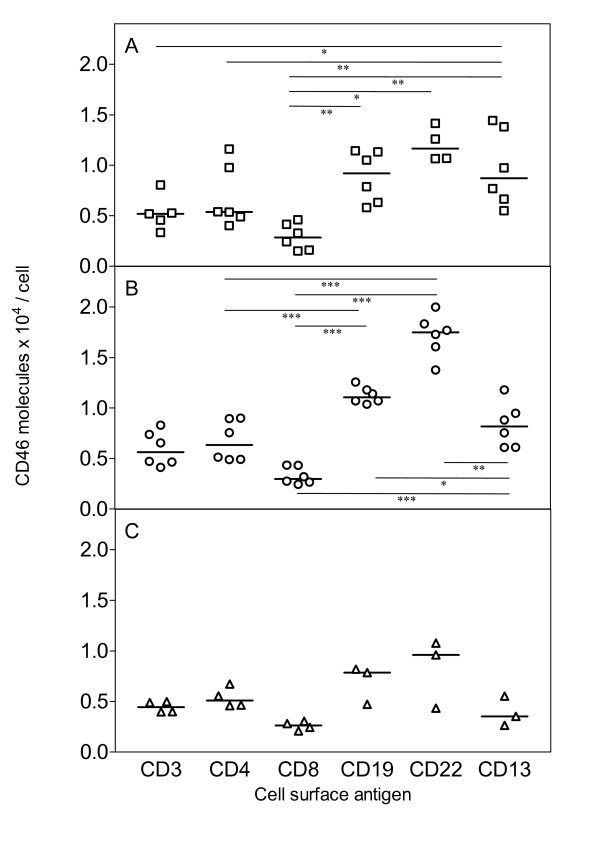
Detection of CD46 molecules on cell membranes of mature leukocytes from cord blood (CB), [A, squares], peripheral blood of adult donors (PB), [B, circles] and leukapheresis products (LP) from G-CSF mobilised precursor cells [C, triangles]. Statistical analysis was performed by paired t-test: *** p < 0.001, ** 0.001<p < 0.01 and * 0.001<p < 0.5. Median values are indicated as horizontal bars.

**Figure 2:**
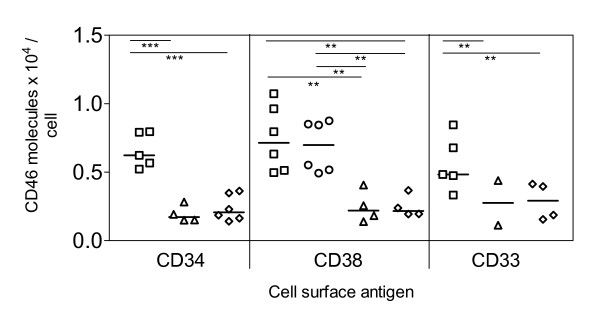
Detection of CD46 molecules on cell membranes of CD34^+^, CD38^+^, CD33^+ ^hematopoietic progenitor cells from cord blood (CB) [squares], peripheral blood of adult donors (PB) [circles], leukapheresis products (LP) from G-CSF mobilised precursor cells [triangles] and MACS sorted CB CD34^+ ^cells [diamonds]. Statistical analysis was performed by unpaired t-test: *** p < 0.001 and ** 0.001<p < 0.01. Median values are indicated as horizontal bars.

Levels of CD46 obtained on T and B-lymphocytes of CB, PB and LP are shown in figure 1. CD46 expression on B-lymphocytes (CD22^+^, CD19^+ ^cells) was significantly higher than on T-lymphocytes from CB and PB. Highest CD46 expression levels were detected on CD22^+ ^B-cells, the median number of molecules per CD22^+ ^cell in CB, PB and LP was 11,650 (range, 10,702–14,158), 17,490 (range, 13,772–19,997) and 9,618 (range, 4,339–10,774), respectively. On CD19^+ ^B-cells the median number of CD46 molecules per cell in CB, PB and LP was 9190 (range, 5,807–11,437), 11,060 (range, 10,378–12,570) and 8,033 (range, 4,718–8,205), respectively. Lowest numbers of CD46 antigen levels were detected on CD8^+ ^T-cells from CB, PB and LP, i.e. 2,851 (range, 1,506–4,604), 2,965 (range, 2,451–4,343) and 2,442 (range, 2,079–3,053), respectively. Expression of CD46 on CD13^+ ^myeloid cells was similar to CD3^+ ^and CD4^+ ^lymphocytes. CD46 antigen expression on CD34^+ ^and CD38^+ ^precursors and CD33^+ ^cells is given in figure 2. Depending on the cell source or on the application of MACS separation, CD46 levels varied considerably. CD34^+^, CD38^+ ^and CD33^+ ^cells from CB expressed significantly more CD46 than corresponding cells from LP or from CB after MACS separation. The median number of CD46 molecules per CD34^+ ^cell in CB, LP and CB after MACS separation was 6,232 (range, 5,219–7,956), 1,715 (range, 1,494–2,822) and 2,074 (range, 1,418–3,621), the number per CD38^+ ^cell was 7,141 (range, 4,975–10,730), 2,195 (range, 1395–4058) and 2,169 (range, 1,945–3,665) and the number per CD33^+ ^cell was 4,828 (range, 3,332–8455), 2,760 (range, 1,128–4,392) and 2,913 (range, 1,552–4,133), respectively. In addition, the T lymphoid cell line CRF-HSB-2 and the myeloid KG-1 cell line expressed 29,245 and 38,141 CD46 molecules per cell.

Our experiments show that mature lymphocytes and myeloid cells, as well as hematopoietic progenitor cells, express CD46. B-lymphocytes express higher levels of CD46 than T-lymphocytes; CD8^+ ^T-lymphocytes exhibit less CD46 than CD4^+ ^lymphocytes. Despite the discovery of HHV-6 as a B-lymphotropic virus, this does not correlate with the common view in the literature that T-lymphocytes would be *in vivo *and *in vitro *the preferred cells for HHV-6 infection [14]. Santoro *et al*. [15] suggested the existence of additional cellular factors, possibly co-receptors that are crucial for HHV-6 infection. However CD34^+ ^and CD38^+ ^hematopoietic progenitor cells from untreated CB express CD46 levels comparable to CD4^+ ^cells. Thus, there is evidence that CD34^+ ^progenitor cells as well as mature leukocytes carry HHV-6 receptors and thereby fulfil the basic requirement for susceptibility to HHV-6 infection.

The CD46 expression level on progenitor cells decreases to approximately one third on CD34^+ ^and CD38^+ ^cells from patients after G-CSF induced stem cell mobilisation and leukapheresis. Similarly, CD46 expression is reduced after immune affinity selection of CD34^+ ^cells by MACS separation. We cannot explain why CD34^+ ^cells in LP and CB after MACS separation bear lower amounts of CD46 than their native, non-manipulated counterparts. We cannot exclude that either *in vitro *manipulations lead to antigen down-regulation, antigen loss, sterical hindrance of antigen recognition, conformational change or that *in vivo *G-CSF treatment leads to a reduction of CD46 expression on the cell membrane. Seya *et al. * [16] showed a decrease of CD46 expression on leukaemia cell lines by *in vitro *G-CSF treatment.

Summing up, our results show significant expression of CD46 on various types of blood leukocytes including hematopoietic progenitor cells. Consequently these cells are fulfilling a requirement for HHV-6 infection. However, the level of expression appears not to be the only criterion for susceptibility to HHV-6.

## Authors' contributions

ST contributed to the sample collection, performed FACS measurements, analysed the results and devised the manuscript.

AR contributed to study design and assisted the experiments as well as data analysis.

AN contributed to study design and mainly revised the manuscript.

WS composed the initial conception and contributed to data interpretation and manuscript revision.

All authors read and approved the final manuscript.
